# Navigating challenges in hydrocephalus following intraventricular hemorrhage: a comprehensive review of current evidence

**DOI:** 10.3389/fneur.2025.1630286

**Published:** 2025-08-18

**Authors:** Haozhou Wang, Xiaofeng Chen, Chao You, Ke Wu, Tong Sun

**Affiliations:** ^1^Department of Neurosurgery, West China Hospital, Sichuan University, Chengdu, Sichuan, China; ^2^Center of Gerontology and Geriatrics, and National Clinical Research Center of Geriatrics, West China Hospital, Sichuan University, Chengdu, Sichuan, China; ^3^Department of Neurosurgery, Xichang People’s Hospital, Liangshan, Sichuan, China

**Keywords:** intraventricular hemorrhage, hydrocephalus, cerebrospinal fluid, erythrocyte lysis, thrombin, ependymal damage, inflammatory response

## Abstract

Hydrocephalus following intraventricular hemorrhage (IVH) is a complex neurological condition resulting from cerebrospinal fluid (CSF) circulation disruptions due to intraventricular blood entry. This review synthesizes current evidence on pathophysiology, mechanisms, and treatment strategies. Following IVH, erythrocyte lysis releases hemoglobin and iron, triggering oxidative stress and ferroptosis, while thrombin activation, TGF-β1-mediated fibrosis, inflammatory cascades, and ependymal damage collectively contribute to ventricular enlargement. Key animal models elucidate roles of oxidative stress, cytokines, and complement activation in IVH-induced injury. We highlight evolving insights into CSF absorption pathways and blood metabolite interactions, alongside persistent clinical challenges including delayed diagnosis and therapeutic limitations. Experimental approaches such as thrombolytics, iron chelators, and inflammatory inhibitors show preclinical potential but face significant translational barriers: thrombolytics reduced mortality in the CLEAR III trial yet failed to improve functional outcomes or shunt dependence; iron chelation lacks robust clinical validation; and TGF-β1 inhibition yields conflicting efficacy across models. Future research must address the interplay of blood components, inflammatory mediators, and structural damage, prioritizing biomarker discovery and rigorously validated therapeutic innovation.

## Introduction

Hydrocephalus is characterized by the abnormal accumulation of cerebrospinal fluid (CSF) within the ventricular system, leading to ventricular enlargement. This condition arises from disruptions in CSF circulation, which may result from obstruction, secretion disorders, or absorption deficiencies (1, 2). The widely accepted theory of CSF circulation posits that approximately 80% of CSF is produced by the choroid plexus within the ventricular system, while the remaining 20% is generated through interstitial fluid transport from brain parenchyma. Following its production, CSF circulates through the ventricular system to the subarachnoid space, where it is reabsorbed into the venous system via arachnoid granulations, thus completing the CSF circulation pathway (3, 4).

Hydrocephalus is classified based on its etiology and mechanism. Mechanistically, it is divided into obstructive (non-communicating) and communicating hydrocephalus. Etiologically, hydrocephalus is categorized into congenital, idiopathic, and acquired forms (5). Clinically, acquired hydrocephalus, often referred to as secondary hydrocephalus, results from a range of neurological disorders such as intracerebral hemorrhage (ICH), subarachnoid hemorrhage (SAH), traumatic brain injury, brain tumors, and intracranial infections.

Among the various neurological conditions that can lead to hydrocephalus, intraventricular hemorrhage (IVH) is a prominent cause (6). IVH is commonly observed in both elderly individuals and neonates (7–10). In adults, IVH often follows spontaneous ICH, aneurysmal SAH, vascular malformations, or traumatic brain injury, which subsequently invade the ventricular system (11). Risk factors for IVH include advanced age, large hematoma volume, and hypertension (12, 13). The occurrence of IVH significantly impacts prognosis and notably increases the risk of developing hydrocephalus (14). Literature indicates that approximately 40% of adults with spontaneous ICH (non-traumatic) experience IVH, with 51–89% of these patients developing secondary hydrocephalus (15). In neonates, IVH frequently arises from germinal matrix hemorrhage (GMH) that affects the ventricular system (GMH-IVH). The germinal matrix is richly vascularized but contains numerous immature, irregular, and fragile capillaries and veins, making it prone to hemorrhage (16). GMH-IVH is most prevalent among extremely low birth weight infants (birth weight <1,500 grams), with approximately 20–30% of these infants developing GMH-IVH and around one-third progressing to hydrocephalus (17).

IVH inflicts direct damage to brain tissue, as well as contributes to both acute and chronic hydrocephalus. The mechanisms underlying secondary hydrocephalus following IVH remain incompletely understood (18). Elucidating these mechanisms is crucial for identifying therapeutic targets to mitigate both primary and secondary damage caused by IVH, thereby improving patient neurological outcomes. This review aims to explore the pathophysiological changes in brain tissue following IVH, key metabolic products, the mechanisms of hydrocephalus development, and current treatment strategies.

## IVH and brain injury

IVH frequently induces a spectrum of primary and secondary brain injuries. Clinicians and researchers frequently employ grading systems to assess the extent of brain damage and prognosis associated with IVH (19). These grading systems consider factors including hematoma volume, location, and ventricular enlargement (20). Clinically, IVH often presents with increased intracranial pressure, varying degrees of consciousness disturbances, neurological deficits, and seizures. The underlying pathophysiological changes are primarily six-fold: periventricular white matter softening; ischemic infarction secondary to hemorrhage; release of harmful substances such as oxygen free radicals and inflammatory cytokines into the CSF circulation; hypotensive and increased intracranial pressure-induced reductions in cerebral perfusion pressure and ischemic damage to periventricular white matter; and the development of hydrocephalus post-ventricular enlargement, which exacerbates oxidative stress, inflammation, and intracranial pressure (19–21). In recent studies, more and more researchers have proved that hydrocephalus should be considered as an independent factor among the factors that cause brain injury. Wahjoeprumono et al. (22) conducted research that assessed the independent influence of hydrocephalus on brain injury. In this study, the researchers compared the mortality and functional outcome between ICH, IVH with hydrocephalus and ICH only (ICH + IVH + hydrocephalus group vs. ICH group). They found that the 30-day and 90-day mortality rate was higher in patients with ICH + IVH + hydrocephalus than in patients who were only diagnosed with ICH only. It’s noted that hydrocephalus leads to higher mortality and morbidity remains elusive, but the exact mechanism has not been explained clearly.

## IVH and ferroptosis

In recent years, more and more studies have shown that neuronal death caused by hydrocephalus is closely related to the mechanism of ferroptosis. Zille, M et al. (23) investigated cell death mechanisms in cultured neurons exposed to hemoglobin or hemin and then compared it with all existing known cell death mechanisms. The researchers found that chemical inhibitors targeting ferroptosis and necroptosis provided protection from hemoglobin- and hemin-induced toxicity. Consistently, molecular markers indicative of both ferroptosis and necroptosis were elevated following ICH in both *in vitro* and *in vivo* settings. Z. Meng et al. (24) explored whether ferroptosis death markers can be used as auxiliary indicators for diagnosis of PHH. Consequently, the researchers revealed the induction of ferroptosis in the choroid plexus of PHH as demonstrated by the excess production of ROS and by lipid peroxidation. Thus, ferroptosis could be a biomarker in the diagnosis of PHH.

## Blood and metabolic products

### Erythrocytes, hemoglobin, and iron

Upon the entry of blood into the ventricular system, erythrocytes begin to lyse within 3 days, releasing substantial amounts of hemoglobin and iron. Numerous studies have demonstrated that both hemoglobin and iron play critical roles in the development and progression of hydrocephalus following hemorrhagic events. Strahle et al. (25) injected hemoglobin and iron into the lateral ventricles of neonatal mice, observing significant ventricular enlargement within 1 day. Similarly, Gao et al. (26) injected lysed erythrocytes and iron into the lateral ventricles of adult rats, resulting in marked ventricular enlargement within 1 day. Additionally, the administration of iron chelators has been shown to significantly reduce the severity of hydrocephalus in various IVH animal models (27). However, the clinical application of iron chelators remains limited, and their efficacy in preventing or mitigating post-IVH hydrocephalus is still debated, lacking robust clinical evidence. Future studies should focus on high-quality basic and clinical research to provide conclusive evidence. Moreover, the presence of macrophages in the ventricular system has been observed to engulf erythrocytes prior to their lysis, resulting in reduced levels of hemoglobin and iron in the CSF (28). Thus, activating phagocytic cells during the early stages of IVH may be a potential strategy to decrease the incidence of long-term hydrocephalus.

### Platelets

Following IVH, platelets release transforming growth factor-beta 1 (TGF-β1), which promotes extracellular matrix protein synthesis and induces subarachnoid space fibrosis. This fibrosis leads to narrowing and obstruction of the subarachnoid space, disrupting CSF circulation and resulting in hydrocephalus (29, 30). In the 1990s, Tada et al. (31) successfully created a communicating hydrocephalus model in mice (10 days old) by injecting recombinant human TGF-β1 into the subarachnoid space, highlighting the significant role of TGF-β1 in hydrocephalus development. TGF-β1 has since been identified as a key biomarker in post-IVH hydrocephalus. Kitazawa et al. (32) reported elevated TGF-β1 levels in CSF following subarachnoid hemorrhage leading to communicating hydrocephalus. Similarly, increased TGF-β1 levels have been observed in the CSF of patients with post-IVH hydrocephalus. However, therapeutic targeting yields conflicting outcomes across models. Table 1 showed the summary of studies on therapeutic targeting TGF-β1 Aquilina et al. (33) found that TGF-β1 inhibition did not reduce ventricular enlargement in neonatal mice post-IVH, whereas Manaenko et al. (34) reported reduced ventricular enlargement and improved cognitive and motor functions with TGF-β1 inhibition in a GMH-IVH model. Conversely, Hoque et al. (35) observed no reduction in ventricular enlargement with TGF-β1 inhibition in a neonatal IVH model, and Yan et al. (36) demonstrated decreased lateral ventricle volume and reduced hydrocephalus incidence with TGF-β1 inhibition in a rat subarachnoid hemorrhage model. These discrepancies highlight TGF-β1’s context-dependent actions: Effective in fibrosis-dominant models (e.g., SAH) but ineffective in developing systems with compensatory inflammation or structural immaturity. Thus, while TGF-β1 is a valid biomarker, its therapeutic utility requires patient stratification.

**Table 1:** Summary of studies on therapeutic targeting TGF-β1.

Study	Model	Intervention	Key finding	Proposed mechanism
Aquilina (2013)	Neonatal IVH mice	TGF-β1 inhibition	No ventricle reduction	Immature arachnoid granulations
Manaengo (2016)	Rabbit GMH-IVH	Anti-TGF-β1	Reduced ventricles & improved function	Fibrosis suppression
Hoque (2016)	Neonatal IVH rat	TGF-β1 blockade	No effect	Compensatory IL-1β upregulation
Yan (2015)	Rat SAH	TGF-β1 inhibition	Decreased hydrocephalus	Ependymal protection

### Thrombin

Upon entering the ventricular system, the coagulation cascade is rapidly activated, resulting in substantial thrombin production. Thrombin has been shown to exacerbate brain injury following intracerebral hemorrhage (ICH) and may also contribute to the formation of hydrocephalus post-IVH (37). Gao et al. (37) demonstrated that thrombin exacerbated ventricular enlargement, disrupted the blood–brain barrier, and damaged the ependymal lining in a rat IVH model. The administration of thrombin inhibitors alleviated these pathological changes. The precise mechanism by which thrombin contributes to post-IVH hydrocephalus remains incomplete. Some studies suggest that thrombin may upregulate TGF-β1 expression, leading to subarachnoid space fibrosis and obstruction, thereby disrupting CSF circulation and causing hydrocephalus (38). Hao et al. (39) recently proposed that thrombin might induce hydrocephalus by downregulating endothelial cadherin in the choroid plexus, with PAR1/p-Src/p-PAK1 pathway inhibition potentially mitigating thrombin-induced hydrocephalus by upregulating endothelial cadherin expression. Future research may explore thrombin inhibition as a potential therapeutic target for post-IVH hydrocephalus.

## Mechanisms of hydrocephalus following IVH

### Blood clots

Early studies commonly attributed hydrocephalus following IVH to blood clots obstructing CSF pathways, particularly the aqueduct of Sylvius and the foramen of Magendie, leading to impaired CSF drainage and ventricular enlargement. Following IVH, blood rapidly disperses through the ventricles and subarachnoid space, forming clots that block CSF circulation and subsequently cause hydrocephalus. This has led to investigations into the use of thrombolytic agents, such as tissue plasminogen activator (tPA) and urokinase-type plasminogen activator (uPA), in the early management of IVH to reduce the incidence of hydrocephalus. Several studies have shown that early administration of tPA and uPA can effectively reduce ventricular enlargement and decrease long-term hydrocephalus rates (40). Similarly, in subarachnoid hemorrhage models, Thomas et al. (41) observed that early thrombolytic therapy could significantly lower the incidence of hydrocephalus. However, the safety and efficacy of thrombolytics in IVH are still debated. Some reports suggest that thrombolytic therapy may worsen edema and induce severe inflammatory responses due to the release of metabolic products from lysed clots (42, 43). A recent multi-center, double-blind, randomized controlled trial (CLEAR III) found that although recombinant tPA reduced mortality at 6 months, it did not improve functional outcomes or reduce the likelihood of shunt placement (44). Thus, while early removal of clots is theoretically a promising approach for preventing hydrocephalus, its clinical adoption is limited by concerns about safety and efficacy, necessitating further high-quality evidence.

### Arachnoid granulations dysfunction

CSF absorption is critical in the development of hydrocephalus following IVH. Traditionally, it is believed that arachnoid granulations, which protrude into the sagittal sinus, are primarily responsible for CSF absorption. Post-IVH, blood and its metabolites can cause fibrosis and inflammation of the arachnoid granulations, obstructing their function and leading to impaired CSF absorption and hydrocephalus (45, 46). However, this model may be incomplete, as arachnoid granulations vary across species, and some, such as rodents and preterm infants, lack these structures (47). Recent research suggests that other potential sites of CSF absorption, including the choroid plexus, pial membrane, lymphatic vessels, and even nasal lymphatics, could also play a role (48). The impact of IVH on these alternative absorption pathways remains under investigation and requires further validation.

### Inflammatory response

Inflammatory responses are pivotal in the pathology of numerous neurological conditions, including trauma and stroke (49–51). IVH triggers significant inflammatory processes, which not only affect disease progression and prognosis but also contribute to the development of hydrocephalus (52–55). Post-IVH, inflammation leads to fibrosis of the arachnoid membrane and ependyma, causing obstruction of CSF pathways and hydrocephalus (50, 56, 57). Additionally, inflammation of the choroid plexus and ependyma has been linked to hydrocephalus. Simard et al. (58) demonstrated that NF-κB activation in these structures was associated with ventricular enlargement in a rat model of IVH. Recent findings suggest that targeting the TLR4-NF-κB pathway may reduce hydrocephalus severity (59). Complement activation, particularly of complement component 3, has also been implicated in post-IVH hydrocephalus. Studies have shown increased levels of complement C3 in the ventricles of rat models, and C3-deficient mice exhibited reduced brain injury and ventricular enlargement post-hemorrhage (60, 61). Comprehensive evaluation of complement inhibition as a therapeutic strategy is still needed.

### Ependymal injury

Ependymal injury is a common consequence of IVH, impacting the functionality of the ependymal lining that lines the ventricles. Ependymal cells, with their cilia, facilitate CSF flow, and their damage can significantly affect CSF dynamics (62). Research indicates that IVH-related damage to ependymal cell junctions, such as N-cadherin, can lead to cell detachment and subsequent atrophy, narrowing, or occlusion of CSF pathways, resulting in hydrocephalus (63, 64). Additionally, ependymal cilia destruction impairs CSF flow and contributes to ventricular enlargement (65, 66).

### Blood–brain barrier disruption

**Figure 1:**
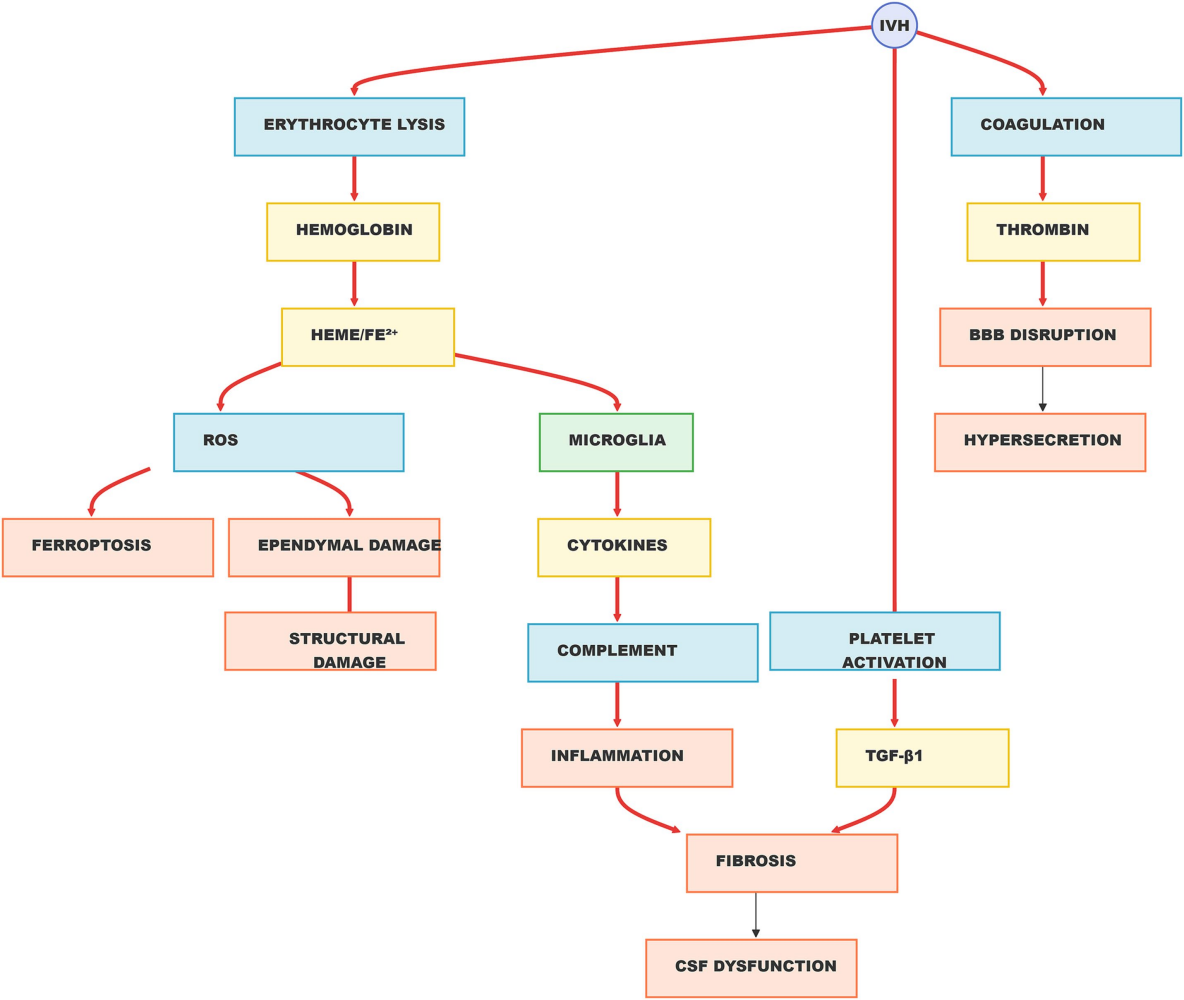
Summary of mechanisms of hydrocephalus following IVH.

The blood–brain barrier (BBB) at the choroid plexus, composed of endothelial cells, basement membrane, pericytes, and astrocyte end-feet, is crucial for maintaining CSF homeostasis. IVH can disrupt this barrier, leading to increased protein levels in the CSF, including TNF-*α*, thrombopoietin, ferritin, TGF-β1, GFAP, and S-100 (67). This disruption can alter osmotic gradients, enhancing CSF secretion and promoting hydrocephalus. Krishnamurthy et al. (68) demonstrated that increased osmotic pressure in the choroid plexus could induce hydrocephalus without obstructing CSF pathways. This supports the theory that IVH-induced BBB damage elevates protein levels in the CSF, disrupting osmotic gradients and contributing to hydrocephalus development (Figure 1).

## Current treatment approaches

### Pharmacological treatment

Pharmacological management typically involves diuretics such as acetazolamide and furosemide, which have been used in hopes of avoiding surgical intervention. However, their clinical efficacy has been suboptimal. Research from the 1990s, including a multicenter, randomized controlled trial, indicated that patients receiving acetazolamide and furosemide had higher rates of disability and a greater likelihood of undergoing shunt surgery compared to those receiving standard treatment (69). Subsequent trials also demonstrated no reduction in shunt implantation rates and an increased risk of disability in patients treated with these diuretics (70). A retrospective study by Kazan et al. (71) corroborated these findings, showing that acetazolamide and furosemide did not lower shunt implantation rates. Consequently, pharmacological treatments are rarely utilized in current clinical practice due to their limited effectiveness and potential to increase disability.

### Surgical treatments

#### Choroid plexus cauterization

Choroid plexus cauterization (CPC) was an early surgical approach for treating hydrocephalus, particularly for communicating hydrocephalus. Although CPC achieved some success, it was associated with high mortality and morbidity rates, and long-term complications remain poorly understood (72, 73).

#### Endoscopic third ventriculostomy

Since the 1990s, ETV has been utilized primarily for obstructive hydrocephalus with patent basal cisterns, where it achieves success rates of 60–80% (74–76). ETV is generally considered successful when there is an obstruction between the third ventricle and the convexity of the brain’s subarachnoid space. However, its efficacy significantly declines in hemorrhage-related cases. Success rates drop to 37–45% in post-IVH hydrocephalus due to arachnoid fibrosis and subarachnoid space obstruction (77). Early failure occurs in >30% of cases within 6 months, often requiring salvage shunting (78), and extensive subarachnoid hemorrhage also reduces the success rate of ETV (75). Recently, ETV has been applied to certain cases of communicating hydrocephalus with favorable outcomes (79). The combination of ETV with CPC has been explored, with some studies suggesting that combined treatment is superior to ETV alone in pediatric hydrocephalus (80). However, other research indicates poor outcomes in preterm infants with IVH undergoing combined ETV and CPC, likely due to multiple obstructions in the CSF pathways (81). ETV remains a situational alternative to shunting, indicated only for obstructive hydrocephalus with radiologically confirmed basal cistern patency. Its limited efficacy in hemorrhage-related cases reflects permanent arachnoid damage that cannot be bypassed by ventriculostomy.

#### CSF shunt surgery

Currently, CSF shunt surgery remains the most common treatments (82). The frequently used shunt types include ventriculoperitoneal shunt (VPS), lumboperitoneal shunt (LPS), and ventriculoatrial shunt (VAS) (74–76, 78–81, 83–85). VPS is widely preferred for both adults and infants, while LPS is used for patients with communicating hydrocephalus following hemorrhage (86). After surgery, most patients experience rapid symptom relief and significant reduction in ventricular size.

#### Endoscopic hematoma evacuation

Endoscopic hematoma evacuation is a minimally invasive surgical evacuation of IVH using neuro-endoscopy, often combined with EVD (87). However, an adequately powered RCT comparing endoscopic hematoma evacuation plus EVD with EVD alone is still lacking and there is also still considerable uncertainty regarding patient selection, intraventricular hematoma volume, ICH volume, timing, and for the surgical procedure itself, including whether to perform septostomy and attempt to clear the contralateral ventricle, or whether to enter the third ventricle (87).

#### Stereotactic hematoma evacuation

Stereotactic techniques rapidly decompress ventricular systems by evacuating clots, restoring CSF dynamics which has a direct impact on hydrocephalus. In a guideline on stroke due to spontaneous ICH (87), there are four RCTs comparing minimal invasive surgical techniques with medical management. Minimal invasive surgery aimed at hematoma evacuation compared with standard medical treatment improved functional outcome (mRS score of 0–3) at 3–6 months (OR 1.84, 95% CI 1.29–2.61) and reduced the risk of death at 1–12 months (OR 0.49, 95% CI 0.30–0.81).

#### Lumber drainage

Lumbar drainage serves as a temporary CSF diversion tool for acute hydrocephalus, particularly in communicating types or as a perioperative adjunct. While it can rapidly alleviate symptoms, its use requires careful patient selection, infection prevention, and transition to permanent solutions to avoid complications. There are lack of trial investigating whether early insertion of a lumbar drainage leading to better functional outcome and reduced shunt dependency (87).

#### Choosing the optimal surgical approach

The optimal surgical approach remains a topic of debate among neurosurgeons. VPS is generally considered the first-line treatment (88). Comparatively, ETV is associated with lower infection rates but higher severe complication and failure rates, leading to limited use in treatment (89–91). LPS avoids direct brain tissue damage, but it has a slightly higher failure and over-drainage rate compared to VPS (92–95). A single-center, retrospective study advocate for LPS as a treatment for adults with post-hemorrhagic communicating hydrocephalus (96). However, there is a lack of randomized clinical trials comparing outcomes among different surgical approaches, highlighting the need for more high-quality, evidence-based research to inform clinical decision-making (97). Table 2 showed the summary of recent studies on clinical outcomes of hydrocephalus following intraventricular hemorrhage.

**Table 2:** Summary of recent studies on clinical outcomes of hydrocephalus following intraventricular hemorrhage.

Study	Patients	Design	Group	Sample size	Conclusion
Kang et al., 2000 (98)	Adults	Retrospective	VPS vs. LPS	32 vs. 22	VPS better than LPS
Koksal et al., 2007 (99)	Infants	Retrospective	VSGS	25	VSGS was safe and effective
Chamiraju et al., 2014 (100)	Infants	Retrospective	ETV + CPC	27	Successful rate: 37%
Chrastina et al., 2018 (79)	Adults	Retrospective	ETV	35	Successful rate: 40%
Wang et al., 2019 (96)	Adults	Retrospective	VPS vs. LPS	102 vs. 56	Equal outcomes
Luther et al., 2020 (101)	Infants	Retrospective	VPS vs. ETV	6,206 vs. 4,810	VPS better than ETV

ETV, endoscopic third ventriculostomy; CPC, choroid plexus cauterization; VPS, ventriculoperitoneal shunt; LPS, lumboperitoneal shunt; VSGS, ventriculosubgaleal shunt.

## Potential treatment strategies for hydrocephalus in the future

*Activation of fibrinolysis and phagocytic Systems*: Accelerating the dissolution of blood clots by enhancing fibrinolytic activity and the brain’s phagocytic response.
*Inhibition of complement-mediated Hemolysis*: Suppressing complement activation to reduce red blood cell breakdown and associated inflammatory responses.
*Enhancement of iron chelation*: Reducing brain iron deposition through chelation therapy to mitigate iron-induced neurotoxicity.
*Maintenance of ependymal cell Function*: Targeting intercellular junction proteins in ependymal cells to reduce damage to cilia and maintain their normal function.
*Inhibition of TGF-β1*: Reducing subarachnoid fibrosis by targeting Transforming Growth Factor-beta 1 (TGF-β1) to prevent excessive scarring and fibrosis.
*Early administration of thrombolytics*: Administering thrombolytic agents early after hemorrhage to promote re-opening of blocked ventricles and subarachnoid spaces.
*Reduction of inflammatory responses*: Mitigating inflammation in the choroid plexus and ependymal lining post-hemorrhage to prevent secondary damage and hydrocephalus.
